# Transcription factor specificity protein 1-mediated Serine/threonine kinase 39 upregulation promotes the proliferation, migration, invasion and epithelial–mesenchymal transition of hepatocellular carcinoma cells by activating the transforming growth factor-β1 /Smad2/3 pathway

**DOI:** 10.1080/21655979.2021.1947939

**Published:** 2021-07-19

**Authors:** Jing Wang, Zhenyu Fan, Jia Li, Jingmao Yang, Xiaofei Liu, Jilin Cheng

**Affiliations:** a Department of Hepatology, Tianjin Institute of Hepatology, Tianjin Second People’s Hospital, Tianjin, China; b Department of Gastroenterology and Hepatology, Shanghai Public Health Clinical Center, Fudan University, Shanghai, China

**Keywords:** Hepatocellular carcinoma, Serine/threonine kinase 39, Smad2/3, specificity protein 1, transforming growth factor β1

## Abstract

Bioinformatics analysis showed that Serine/threonine kinase 39 (STK39), which was testified to play an important role in human cancers, may be a hub gene in diagnosing hepatocellular carcinoma (HCC). This study aimed to explore whether STK39 could be regulated by specificity protein 1 (SP1) to affect HCC cells malignant processes. Firstly, STK39 expression in tissues of HCC patients and several cell lines was analyzed. After STK39 silencing, cell proliferation was evaluated by methyl thiazolyl tetrazolium and colony formation assay. Tunel staining was used to detect cell apoptosis. Then, the abilities of cell migration and invasion were determined with wound healing and transwell assays. The expression of epithelial–mesenchymal transition (EMT)-related proteins and transforming growth factor-β1 (TGF-β1)/Smad2/3 pathway proteins was tested by western blot analysis. Thereafter, cells were overexpressed with SP1 under the circumstance of STK39 knockdown, and then the above cellular processes were under observation. Results revealed that the increased expression of STK39, which was found in both HHC patients and HCC cell lines, exhibited poor HCC prognosis. STK39 silencing inhibited Hep3b cell proliferation, migration, invasion, EMT and TGF-β1/Smad2/3 expression but promoted cell apoptosis. Additionally, SP1 could bind to the STK39 promoter and facilitate STK39 expression. Further studies revealed that the effects of STK39 silencing on Hep3b cells were blocked by SP1 overexpression. In conclusion, SP1-mediated STK39 up-regulation leads to the increased proliferation, migration, invasion and EMT of HCC cells via activating TGF-β1/Smad2/3 pathway. Therapies that target SP1 to knockdown STK39 expression may contribute to the inhibition of HCC progression.

## Graphical abstract

**Figure:**
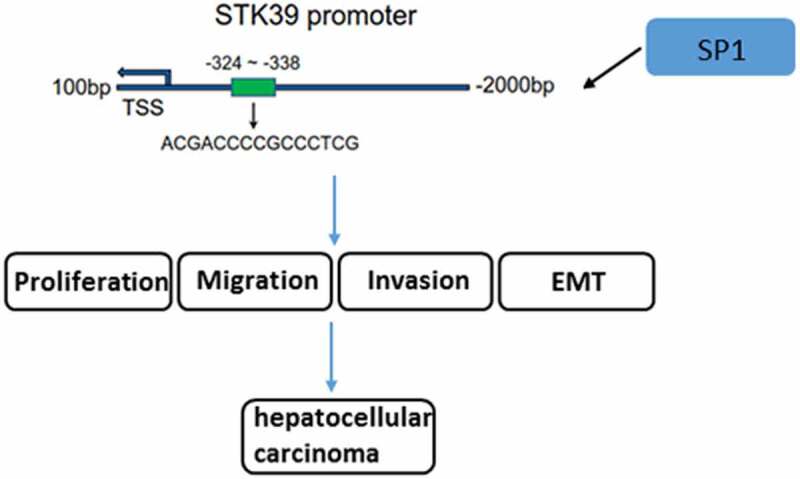

## Introduction

Hepatocellular carcinoma (HCC) is a kind of digestive system tumors familiar to the public. Globally, the incidence of HCC ranks sixth in tumor incidence and remains the fourth most common fatal cancers leading to a high rate of mortality [1]. As diagnostic and therapeutic techniques evolve, it has been found that the prognosis of HCC patients has been greatly improved. The 5-year survival rate of early HCC patients who received radical resection was 70% [2]. However, more than 70% of patients underwent tumor recurrence or metastasis 5 years after radical resection of HCC [3]. As a matter of fact, the result of prognosis of HCC is not as optimally as desired. The main reasons include hidden onset, high malignancy, and easy recurrence and metastasis [4]. Therefore, studying the molecular mechanism of the malignant development as well as the recurrence and metastasis of HCC may provide a theoretical basis for finding effective predictors and potential therapeutic targets. What’s more, it also has great clinical significance for diagnose and treatment in an early stage.

The genes associated with the development of HCC are widely screened. Recently, Serine/threonine kinase 39 (STK39, also known as SPAK/PASK) was identified as one of the hub genes by means of the diagnose of HCC in the TCGA database. Immune infiltration analysis and GSEA enrichment analysis suggested that STK39 was correlated with the immune cell infiltration, involving many mechanisms like angiogenesis and epithelial–mesenchymal transition (EMT) [5]. STK39, a Ser/Thr kinase, has been widely considered to activate MAPK p38 pathway, thus participating in stress response [6]. In recent years, a growing body of literature has shown the roles of STK39 in human cancers. For instance, the therapeutic resistance of breast cancer is affected by down-regulated levels of STK39 [7]. The overexpression of STK39 was found in osteosarcoma and renal cell carcinoma (RCC). STK39 knockdown exerted its inhibitory effects on the proliferation of osteosarcoma cell and RCC cells [8,9]. A recently study has demonstrated that STK39 is a novel kinase contributing to the progression of HCC [10]. However, the upstream regulatory mechanisms of STK39 in HCC still remain to be elucidated.

STK39 was predicted to be targeted by the transcription factor specificity protein 1 (SP1) using JASPAR database (http://jaspar.genereg.net/). SP1, a member of SP family, has been proved to regulate cell growth, apoptosis and carcinogenesis to a large extent [11]. In fact, most of the SP1-target genes are key players in cell proliferation and oncogenesis, including prominent oncogenes and tumor suppressors. As a result, SP1 is considered as a long-standing target in cancer chemotherapy [12]. Notably, SP1 has been implicated to be involved in the progression of HCC [13,14], but the mechanisms remain to be investigated.

In this study, we speculated that SP1 may regulate HCC cells proliferation, migration, invasion and EMT via binding to STK39 promotor. The aim is to understand whether STK39 could regulate HCC progression after being targeted by SP1, as well as uncover the potential pathways involved in. Our findings might identify a new theoretical basis for targeted therapy for HCC.

## Materials and methods

### Cell culture

Human normal HHL-5 hepatocytes were purchased from ATCC and cultured in DMEM-F12 medium (1:1; Invitrogen) supplemented with 10% fetal bovine serum (FBS; Gibco). The human HCC cell lines (MHCC97-H, SK-Hep-1, Huh-7 and Hep3b) were obtained from National Collection of Authenticated Cell Cultures (Shanghai, China). The culture medium for MHCC97-H and Huh-7 was DMEM (Invitrogen) containing 10% FBS. The culture medium for SK-Hep-1 and Hep3b was MEM (Gibco) containing 10% FBS. All cells were cultivated at 37°C containing 5% CO_2_ and 95% air.

### Cell transfection

The design and synthesis of the short hairpin RNA (shRNA) targeting STK39 (shRNA-STK39#1 and shRNA-STK39#2), and scrambled negative control shRNA (sh-NC) were generated by GenScript Co., Ltd, (Nanjing, China). The full length of SP1 was designed and cloned into pcDNA3.1 vector by Gene Script Biotech Co., Ltd. (Nanjing, China). The empty vector pcDNA3.1 was actually considered as negative control (NC). The cells were cultivated overnight to reach 60–70% confluence before transfection. The transfection procedure was conducted using Lipofectamine 2000 (Invitrogen) according to standard protocol and cells were chosen for subsequent experiments at 48 h post-transfection.

### Cell viability assay

The detection of cell viability was conducted by means of methyl thiazolyl tetrazolium (MTT; Beyotime, China). Briefly, the control or transfected Hep3b cells (2x10^3^/well) were plated in 96 well-plate and cultured for 24, 48 or 72 h, and then each well was added with 10 μL MTT working solution, followed by incubation with normal cell culture medium for 4 h. Subsequently, samples were treated with 100 μL formazan until all the purple crystals have dissolved (3–4 h). Finally, the absorbance was detected at 570 nm.

### Colony formation

Hep3b cell suspension was resuspended in 1 ml medium during the experiment of colony formation assays. Then, the cells were plated in a 24-well plate and incubated for 2 weeks. Crystal violet was employed to stain the cells. The colony that was over 50 cells was considered as a positive colony (magnification, x100).

### Wound healing assay

Hep3b cells were inoculated in a 6-well plate (4x10^4^ cells/well). When the confluence was up to 70–80%, a wound on the cell surface was formed by a pipette tip. An inverted microscope (Olympus Corporation) was adopted to observe the cells during the experiments and the images were labeled as 0 h. Subsequently, the medium was taken place of serum-free medium, in which a more detailed cell culture was performed for 24 h. An inverted microscope (Olympus Corporation) was utilized to photograph the cell migration.

### Transwell assay

Transwell assay was evaluated using 24-well culture plates with 8-μm pore inserts (Transwell; Falcon, BD Biosciences). The lower chamber was filled with 600 µL DMEM containing 10% FBS. A serum-free medium containing 4 × 10^4^ Hep3b cells covered the upper chamber. After 24 h of incubation, the migrated cells were fixed with 4% methanol. Those invasive cells stained with 0.1% crystal violet were carefully counted by a counting chamber.

### Tunel staining

Cell apoptosis was detected using Tunel assay kit (Beyotime, China) in accordance with the manufacturer’s protocol. The normal Hep3b cells or transfected cells were fixed with 4% paraformaldehyde and then treated with PBS containing 0.3% Triton X-100. After being exposed to fluorescein (green)-labeled dUTP solution in the dark for 1 h, the images were captured using a fluorescent microscope (Olympus, Japan).

### Dual-luciferase report assay

The amplification and cloning of the wide type (WT) or mutant (MUT) STK39 sequences synthesized by Shanghai GenePharma co., ltd (Shanghai, China) occurred in the downstream of the luciferase reporter gene in pMIR-REPORT luciferase vectors (Thermo Fisher Scientific, Inc.). Then, the cells were co-transfected with pcDNA3.1-SP1 or empty pcDNA3.1. After transfection for 48 h, the luciferase activities were examined using a dual-luciferase reporter system (Promega Corporation).

### Chromatin immunoprecipitation (ChIP) assay

ChIP assays were conducted in accordance with the manufacturer’s guidelines. Hep3b cells at the density of 4 × 10^7^ were washed with cold PBS and then crosslinked with 1% formaldehyde. Subsequently, the cells were subjected to ChIP assay with a High-Sensitivity Kit (Abcam). The antibodies used in this assay including anti-SP1 (5 µg for 25 µg of chromatin; cat. no. ab231778; Abcam) and IgG (negative control). The primer sequences of quantitative polymerase chain reaction (qPCR) assay for detecting STK39 level were forward 5’-GTGGAGTGACGACGAGATGG-3’, reverse 5’- CATTGGGTGGGCCCTCTG-3’.

### Reverse transcription-qPCR (RT-qPCR) analysis

The extraction of total RNA was carried out by means of TRIzol reagent (Invitrogen). Afterward, a total of 5 μg RNA was reversely transcripted into cDNA using TaqMan one-step reverse transcription (Applied Biosystems, USA). PCR was conducted on an ABI 7500 instrument (Applied Biosystems) by means of iTaq™ Universal One-Step iTaq™ Universal SYBR® Green Supermix (Bio-Rad Laboratories, Inc.) to analyze the levels of gene expression. The specific primers for STK39 and SP1 were listed as follows:

STK39, forward, 5ʹ-GTGGAGTGACGACGAGATGG-3ʹ, reverse, 5ʹ- CATTGGGTGGGCCCTCTG-3ʹ;

SP1, forward, 5ʹ-CCCTTGAGCTTGTCCCTCAG-3ʹ, reverse, 5ʹ-GTAGCCCCAGAGGAGGAAGA-3ʹ;

GAPDH, forward, 5ʹ-GCAACCGGGAAGGAAATGAATG-3ʹ, reverse, 5′-CCCAATACGACCAAATCAGAGA-3ʹ. The calculation of gene expression was performed in a way of fold change based on the 2^−ΔΔCq^ method [15]. Glyceraldehyde-phosphate dehydrogenase (GAPDH) was served as an internal control.

### Western blot analysis

The extraction of total protein in Hep3b cells was carried out by means of RIPA lysis buffer (Beyotime). A bicinchoninic acid protein assay kit (Beyotime, Beijing, China) was adopted for the measurement of protein concentration. Afterward, 10% sodium dodecyl sulfate-polyacrylamide gel electrophoresis (SDS-PAGE) was added into equal amount of proteins (20 μg/lane). Those proteins were later transferred to PVDF membranes which was blocked by 5% nonfat milk. Primary antibodies were employed to facilitate the incubation of membranes at 4^°^C overnight. Primary antibodies against STK39 (1:5000), Bax (1:5000), Bcl-2 (1:2000), matrix metalloproteinase (MMP)2 (1:1000), MMP9 (1:1000), Vimentin (1:2000), E-cadherin (1:10000), N-cadherin (1:10000), TGF-β1 (1:1000), phosphorylated (p)-Smad2 (1:1000), Smad2 (1:1000), p-Smad3 (1:2000), Smad3 (1:5000), SP1 and GAPDH (1:10000) were provided by Abcam. Additionally, the conjugation of secondary antibody and horseradish peroxidase (1:5,000; Abcam) was used to examine the blots. The visualization of protein bands was performed with the aid of an enhanced chemiluminescence substrate (Pierce, USA) using chemiluminescence imaging equipment (Claremont, CA, USA). The relative intensity of target bands was semi-quantified using ImageJ software and normalized by the intensity of GAPDH.

### Statistical analysis

Experimental results were presented in triplicate in the form of the mean ± standard deviation of data. A one-way analysis of variance was adopted to present the comparisons among multiple groups. Meanwhile, Tukey’s test was also performed. Value of P < 0.05 was considered to be of statistically significance.

## Results

### STK39 was up-regulated in HCC patients and cell lines, and predicts poor prognosis

**Figure 1.**
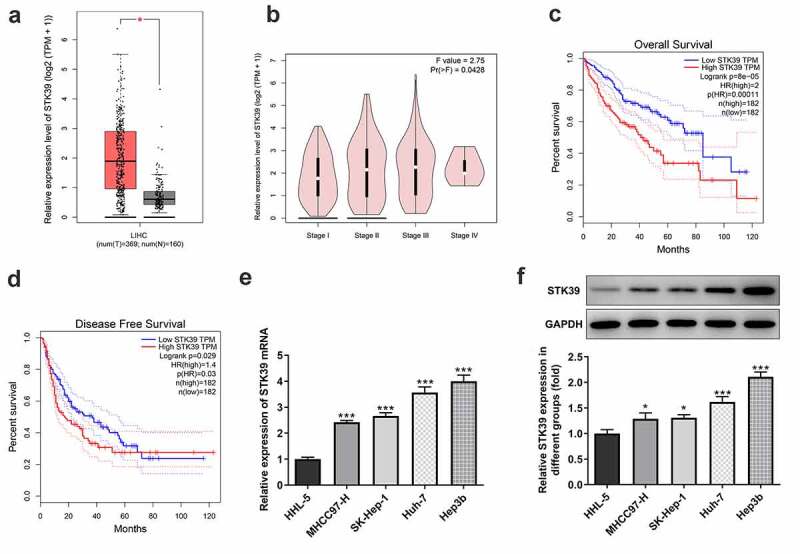
STK39 was up-regulated in HCC and predicts poor prognosis

STK39, a Ser/Thr kinase, has been reported to participate in the progression of multiple human cancers, such as osteosarcoma, renal cell carcinoma and non-small cell type lung cancer cells [8,9,16]. To figure out the possible role of STK39 in HCC, we analyzed the expression of STK39 in HCC using GEPIA database. Figure 1(a) showed that STK39 was highly expressed in the tissues of HCC patients. As Figure 1(b) revealed, STK39 expression was correlated with tumor pathological stage. Meanwhile, the high expression of STK39 predicted the low overall survival (OS) and disease-free survival (DFS) (Figure 1(c,d)). Consistently, compared with normal HHL-5 hepatocytes, HCC cell lines including MHCC97-H, SK-Hep-1, Huh-7 and Hep3b, exhibited significantly higher expression of STK39 mRNA and protein (Figure 1(e,f)). Hep3b was chosen for the following experiments.

(a) The expression of STK39 in liver hepatocellular carcinoma (LIHC, right group) and normal (left group) tissues was analyzed using GEPIA database. *P < 0.05. (b) The expression of STK39 in different pathological stages of HCC was analyzed using GEPIA database. (c,d) The correlation between STK39 expression and overall survival or disease-free survival of HCC was analyzed using GEPIA database. (E-F) The mRNA and protein expression of STK39 in human normal HHL-5 hepatocytes and human HCC cell lines including MHCC97-H, SK-Hep-1, Huh-7 and Hep3b was tested by RT-qPCR and western blot analysis. *P < 0.05 and ***P < 0.001 vs HHL-5.

### STK39 silencing inhibited HCC cells proliferation, migration, invasion, EMT and TGF-β1/Smad2/Smad3 signaling expression

**Figure 2.**
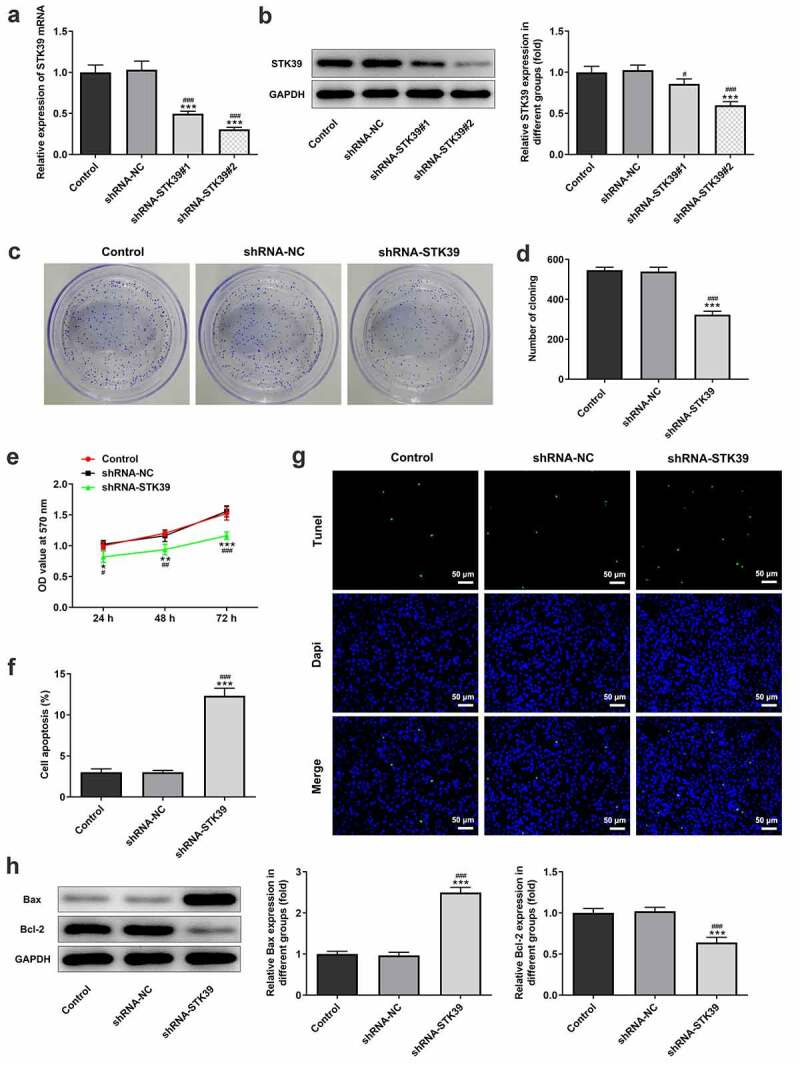
STK39 knockdown inhibited proliferation and induces apoptosis of Hep3b cells

**Figure 3.**
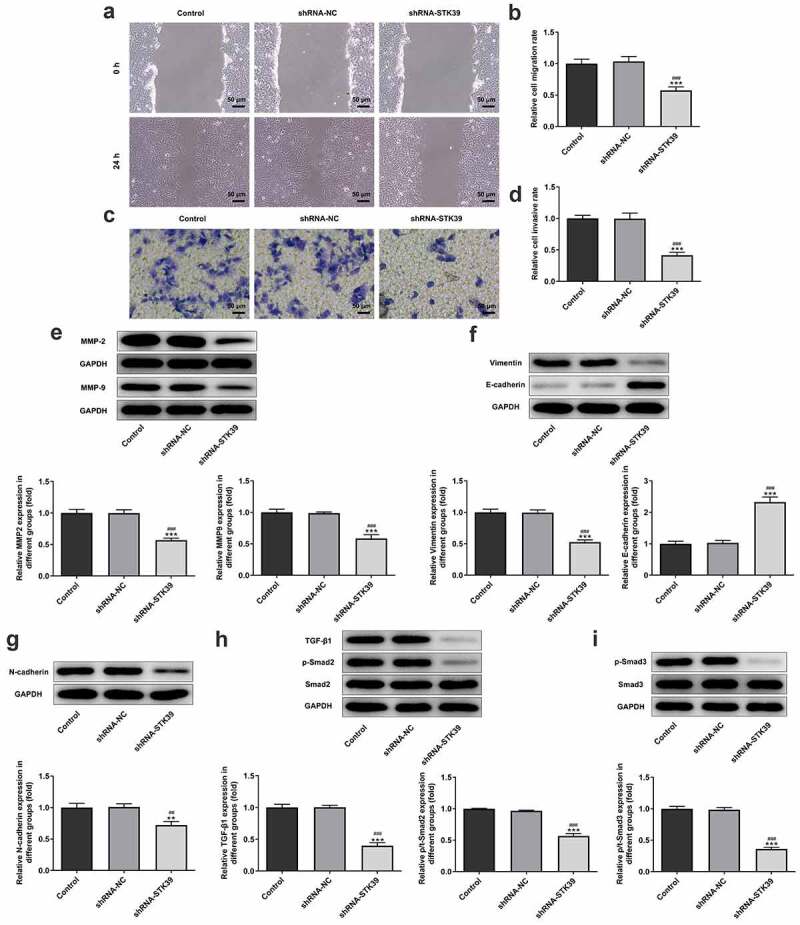
STK39 knockdown suppressed migration, invasion, EMT and TGF-β1/Smad2/3 signaling of Hep3b cells

-To clarify the effects of STK39 on the progression of HCC, TK39 was silenced by transfection with shRNAs and shRNA-STK39#2 was chosen for the subsequent experiments due to its optimal efficacy (Figure 2(a,b)). Figure 2(c,d) implicated that the number of colony formation was obviously reduced upon STK39 knockdown. Besides, STK39 silencing greatly decreased the cell viability of Hep3b cells in comparison with the control group (Figure 2(e)). The alteration of cell apoptosis was also studied. Figure 2(f,g) clearly depicted that the ratio of Tunel-positive (apoptotic) cells was remarkably increased in STK39 silencing cells, indicating that STK39 silencing could promote the apoptosis in Hep3b cells. Consistently, STK39 silencing resulted in increased levels of pro-apoptotic protein Bax but decreased levels of anti-apoptotic protein Bcl-2 (Figure 2(h)). Wound healing and transwell assays were carried out to explore Hep3b cells migration and invasion. Figure 3(a–d) depicted that STK39 knockdown inhibited Hep3b cells migration and invasive rate compared with the shRNA-NC group. In addition, the expression of MMP-2 and MMP-9 was markedly reduced by STK39 knockdown (Figure 3(e)). Moreover, the expression of vimentin, E-cadherin and N-cadherin was measured to reflect EMT process. Results from Figure 3(f,g) revealed that STK39-downregulation increased E-cadherin expression as well as decreased the expression of vimentin and N-cadherin, suggesting that STK39 knockdown inhibited EMT of Hep3b cells. Moreover, STK39 silencing suppressed TGF-β1/Smad2/3 signaling, as evidenced by the considerably down-regulated expression of TGF-β1, p-Smad2 and p-Smad3 in STK39 silencing cells (Figure 3(h,i)).

(a,b) The mRNA and protein expression of STK39 in control Hep3b cells or cells that transfected with indicated shRNAs was determined with RT-qPCR and western blotting. ***P < 0.001 vs Control; ^#^P < 0.05 and ^###^P < 0.001 vs shRNA-NC. (c,d) Colony formation assay was performed to observe cell proliferation. (e) Cell viability at 24, 48 and 72 h was measured respectively by means of MTT assay. (f,g) Cell apoptosis was observed using Tunel staining (×200); (h) The protein expression of Bax and Bcl-2 was detected via western blot assay. *P < 0.05, **P < 0.01, ***P < 0.001 vs Control; ^#^P < 0.05, ^##^P < 0.01 and ^###^P < 0.001 vs shRNA-NC.

(a,b) Hep3b cells were transfected with shRNA-NC or shRNA-STK39 or not, then cell migration was assessed using wound healing assays. (c-d) Cell invasion was evaluated with transwell assay. (e) The protein expression of MMP2 and MMP9 was detected using western botting. (f,g) The expression of EMT-related proteins including vimentin, E-cadherin and N-cadherin was assessed by means of western blot assay. (h) TGF-β1, p-Smad2/Smad2 and p-Smad3/Smad3 expression was detected using western blot. **P < 0.01 and ***P < 0.001 vs Control; ^##^P < 0.01 and ^###^P < 0.001 shRNA-NC.

### SP1 could bind to STK39 promotor and up-regulate STK39 expression

**Figure 4.**
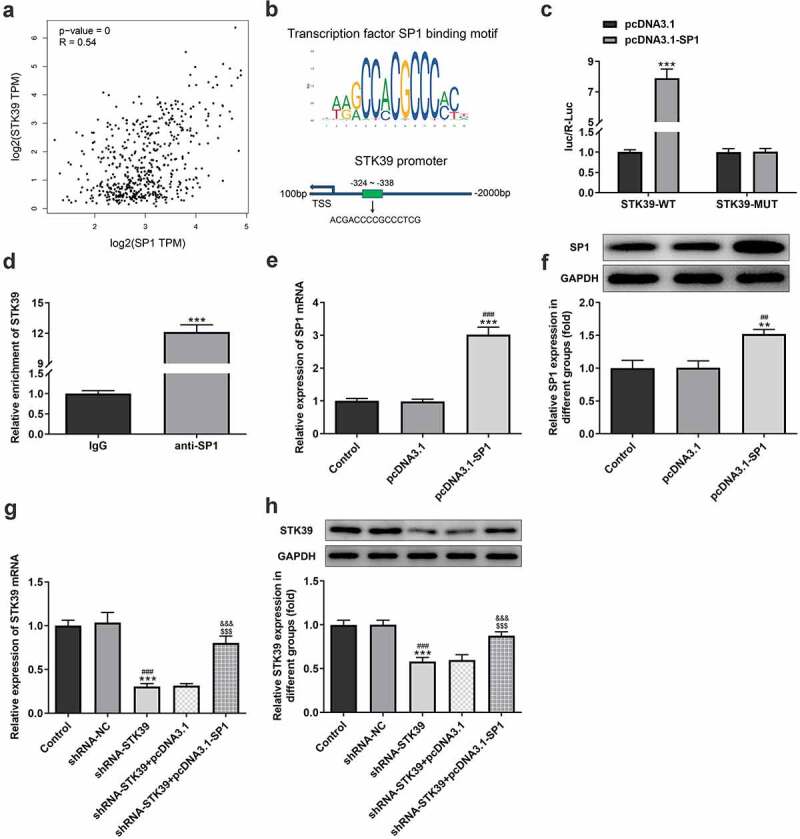
The relationship between SP1 and STK39

To explore the possible regulatory mechanisms of STK39 in the progression of ACC cells, experiments were performed to determine whether SP1 was involved in the effect of STK39 on HCC. We studied the relevance between SP1 and STK39 expression in HCC using GEPIA database, and found that the expression of transcription factor SP1 is positively correlated with that of STK39 (Figure 4(a)). Furthermore, JASPAR database predicted that SP1 could bind to the promoter of STK39 (Figure 4(b)). The interaction between SP1 and STK39 was further validated by dual-luciferase report (Figure 4(c)) and ChIP (Figure 4(d)) assays. Then, we overexpressed SP1 in Hep3b cells using transfection of pcDAN3.1-SP1 (Figure 4(e,f)), and found that cells that co-transfected with shRNA-STK39 and pcDNA3.1-SP1 exhibited significantly higher STK39 expression compared with the shRNA-STK39 group (Figure 4(g,h)), indicating that SP1 could up-regulate STK39 expression.

(a,b) The expression correlation and binding sequences between SP1 and STK39 was analyzed using GEPIA database. (c) The result of dual-luciferase report assay. ***P < 0.001 vs STK39-WT + pcDNA3.1. (d) Chromatin immunoprecipitation (ChIP) assay was adopted to detect the interaction between STK39 and SP1. ***P < 0.001 vs IgG. (e-f) The transfection efficiency of SP1 overexpression in Hep3b cells. **P < 0.01 and ***P < 0.001 vs Control; ^##^P < 0.01 and ^###^P < 0.001 vs pcDNA3.1. (g,h) The mRNA and protein expression of STK39 in Hep3b cells that transfected with indicated vectors was examined with RT-qPCR and western blot assay. ***P < 0.001 vs Control; ^###^P < 0.001 vs shRNA-NC; ^$$$^P < 0.001 vs shRNA-STK39; ^&&&^P < 0.001 vs shRNA-STK39+ pcDNA3.1.

### SP1 overexpression reversed the inhibitory effects of STK39 silencing on the proliferation, migration, invasion and EMT of HCC cells

**Figure 5.**
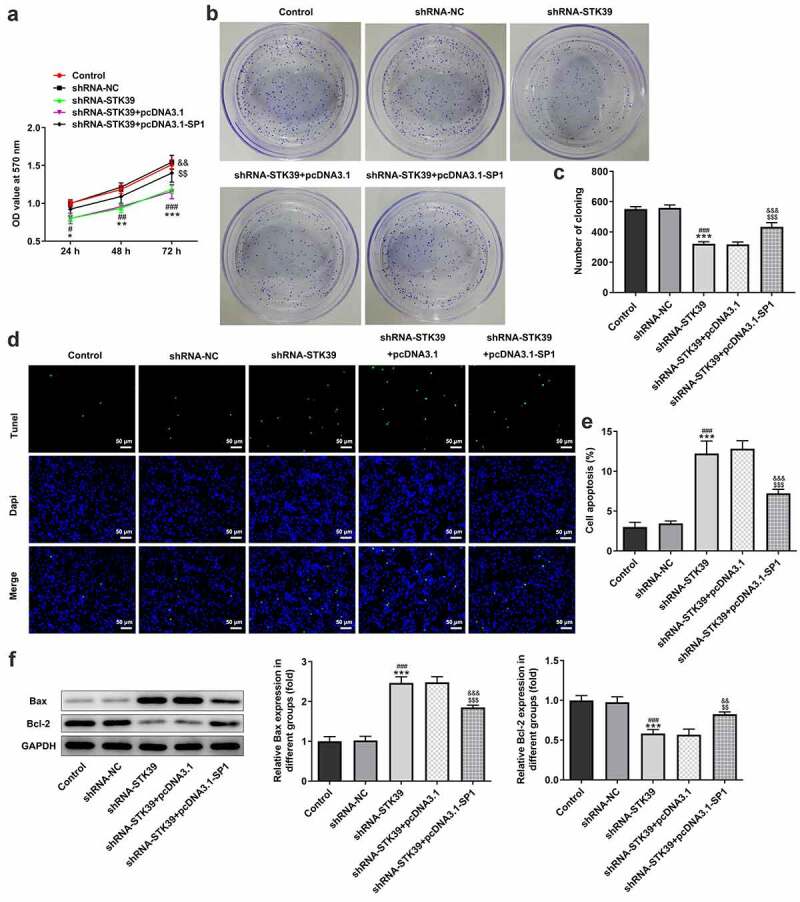
SP1 overexpression blocked the effect of STK39 knockdown on Hep3b cells proliferation and apoptosis

**Figure 6.**
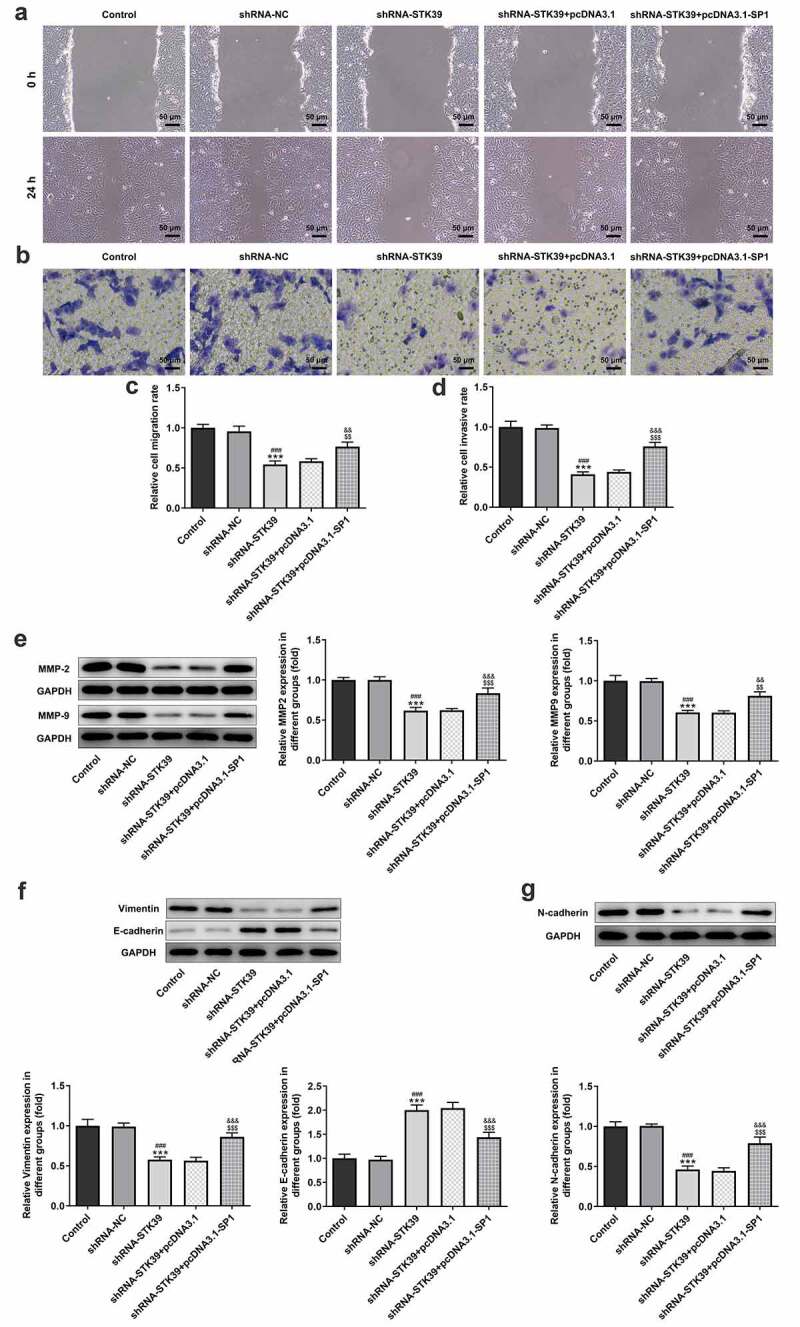
SP1 overexpression alleviated the effect of STK39 knockdown on Hep3b cells migration, invasion and EMT

Finally, to validate our hypothesis that transcription factor SP1-mediated STK39 upregulation promotes the progression of HCC cells, we co-transfected shRNA-STK39 and pcDNA3.1-SP1 into cells to overexpress SP1, and then evaluated the effects of STK39 silencing on HCC cells proliferation, migration, invasion and EMT. Figure 5(a–c) revealed that the STK39 silencing cells with SP1 overexpression exhibited higher cell viability and colony formation than STK39 silencing cells, suggesting that the inhibitory effect of STK39 knockdown on cell proliferation was reversed by SP1 overexpression. SP1 overexpression also inhibited the induction of apoptosis caused by STK39 knockdown, as evidenced by the decreased cell apoptosis ratio and Bax expression (Figure 5(d,f)). However, Bcl-2 expression was increased by SP1 overexpression in the presence of STK39 knockdown compared with cells that only silenced with STK39. Figure 6(a–e) described that SP1 overexpression, to a large extent, remarkably attenuated the inhibitory effects of STK39 knockdown on cell migration, invasion along with MMP-2 and MMP-9 expression. Besides, the reduced expression of vimentin and N-cadherin and enhanced E-cadherin expression caused by STK39 knockdown were markedly reversed by SP1 overexpression (Figure 6(f,g)).

(a) Cell viability of Hep3b cells that transfected with indicated vectors was evaluated by MTT assay. (b,c) Representative colony formation assay for Hep3b cells that transfected with indicated vectors. (d,e) Representative images and quantitative analysis for Tunel staining in Hep3b cells that transfected with indicated vectors (×200). (f) The proteins expression of Bax and Bcl-2 in Hep3b cells that transfected with indicated vectors was tested using western blot analysis. *P < 0.05, **P < 0.01, ***P < 0.001 vs Control; ^#^P < 0.05, ^##^P < 0.01 and ^###^P < 0.001 vs shRNA-NC; ^$$^P < 0.01 and ^$$$^P < 0.001 vs shRNA-STK39; ^&&^P < 0.01 and ^&&&^P < 0.001 vs shRNA-STK39+ pcDNA3.1.

(a,d) Cell migration and invasion were respectively assessed using wound healing assay and transwell assay. (e,g) The protein expression of MMP2, MMP9, vimentin, E-cadherin and N-cadherin were detected using western blot assay. ***P < 0.001 vs Control; ^###^P < 0.001 vs shRNA-NC; ^$$^P < 0.01 and ^$$$^P < 0.001 vs shRNA-STK39; ^&&^P < 0.01 and ^&&&^P < 0.001 vs shRNA-STK39+ pcDNA3.1.

## Discussion

The main point of the current study is to explore STK39 expression in HCC tissues and cell lines. STK39 knockdown suppressed proliferation, migration, invasion and EMT, but induced apoptosis of Hep3b cells. Protein levels related to proliferation, migration, invasion and EMT were strongly influenced by the loss-function of STK39. Furthermore, STK39 knockdown suppressed the expression of TGF-β1 and phosphorylation of Smad2/3. Mechanistically, the binding of SP1 to STK39 promoted STK39 expression, and SP1 upregulation blocked the effects of STK39 silence on cells proliferation, apoptosis, migration, invasion and EMT. Therefore, our data suggested that STK39 may be positively regulated by SP1 and its role in the development of HCC may act as an oncogene.

The abnormal expression of STK39 in various tumors has been proposed. Besides, the abnormal expression of STK39 also affects the progression of cancers. The lower level of STK39 mRNA expression in patients with primary prostate cancer had a higher incidence of metastases [17]. In breast cancer, there was a relevance between the decreased STK39 and treatment resistance [7]. By contrast, it is reported that STK39 was overexpressed in renal cell carcinoma [9], osteosarcoma [8] and breast cancer cells [18], while the knockdown of STK39 inhibited proliferation, metastasis and EMT of these cancer cells. Currently, Zhang et al demonstrated that STK39 is a novel kinase contributing to the progression of HCC [10]. However, the regulatory mechanisms of STK39 upstream in the progression of HCC still remain to be elucidated. A previous study identified STK39 as one of the hub genes in diagnosing HCC, revealing that STK39 has a potential function in HCC [5]. In this study, we compared STK39 expression in liver hepatocellular carcinoma tissues, normal tissues, multiple HCC cell lines and normal HHL-5 hepatocytes. The consistent result was achieved, that is, STK39 was upregulated in HCC tissues and cell lines. Furthermore, the high expression of STK39 is correlated with advanced tumor pathological stage, low OS and DFS. Therefore, STK39 may probably act as an oncogene which regulates the development and progression of HCC.

We silenced STK39 expression in Hep3b cells to further explore the specific role of STK39 in HCC. The results showed that STK39 knockdown reduced cell proliferation, inhibited migration, invasion, MMP2 and MMP9 expression, and downregulated vimentin and N-cadherin expression. However, the apoptosis of Hep3b cells as well as E-cadherin expression were increased by STK39 knockdown. MMPs, containing MMP2 and MMP9, exerted an essential role in metastasis by means of the degradation of extracellular matrix proteins [19]. We also found that the decrease of epithelial markers (i.e., E-cadherin) and increase of mesenchymal markers (i.e., N-cadherin and vimentin) contribute to epithelial tumor cells EMT [20]. Our results revealed the effects of STK39 silencing on HCC cells progression.

TGF-β exhibited its inhibitory effects on tumor in an early stage of cancer via suppressing cell cycle progression and accelerating apoptosis. On the contrary, TGF-β promoted the progression of tumor as well as increased the invasiveness and metastasis of tumor [21]. Upon interacting with its receptors, TGF-β1 resulted in the phosphorylation of Smad2 and Smad3, ultimately causing targeted genes transcription [22]. In breast cancer, TGF-β1/Smad2/3 signaling has been testified to promote the malignant progression of cancers cells [23,24]. Importantly, research has proposed that

TGF-β1 plays key roles in modulating HCC aggressiveness through triggering the EMT of the cancer cells [25]. Activation the TGF-β/Smad signaling pathway can promote the migration and invasion of HCC [26]. In this study, the levels of TGF-β1 and phosphorylation of Smad2 and Smad3 were suppressed in STK39 knockdown cells. This finding was consistent with previous reports [8]. Therefore, the function of STK39 may be recognized as an oncogene due to its partial activation of TGF-β1/Smad2/3 pathways in HCC.

After searching JASPAR database (http://jaspar.genereg.net/), we found that transcription factor SP1 could bind to STK39 promoter, and further studies will be done to confirm this prediction. Moreover, SP1 could positively regulate STK39 expression. SP1 was considered to be involved in the progression of HCC [13,14], therefore we speculated that SP1 may target STK39 to promote STK39 transcriptional expression, thus exerting its effects on HCC. We overexpressed SP1 and verified that SP1 overexpression blocked the antitumor effects of STK39 silencing on Hep3b cells. Therefore, the conclusion can be drawn that it is SP1 that mediated STK39 expression to promote HCC progression by activating TGF-β1/Smad2/3 pathway.

## Conclusions

To sum up, we discovered that STK39 was highly expressed in HCC. SP1-mediated STK39 upregulation led to the elevated proliferation, migration, invasion and EMT of HCC cells via activating TGF-β1/Smad2/3 signaling pathway. Our findings provide evidence for a new regulatory mechanism consisting of SP1 and STK39 in HCC, identifying a new theoretical basis for targeted therapy.

## Data Availability

The datasets generated and/or analyzed during the current study are available on reasonable request.
